# Systematic Review and Consensus Guidelines for Environmental Sampling of *Burkholderia pseudomallei*

**DOI:** 10.1371/journal.pntd.0002105

**Published:** 2013-03-21

**Authors:** Direk Limmathurotsakul, David A. B. Dance, Vanaporn Wuthiekanun, Mirjam Kaestli, Mark Mayo, Jeffrey Warner, David M. Wagner, Apichai Tuanyok, Heiman Wertheim, Tan Yoke Cheng, Chiranjay Mukhopadhyay, Savithiri Puthucheary, Nicholas P. J. Day, Ivo Steinmetz, Bart J. Currie, Sharon J. Peacock

**Affiliations:** 1 Department of Tropical Hygiene, Faculty of Tropical Medicine, Mahidol University, Bangkok, Thailand; 2 Mahidol-Oxford Tropical Medicine Research Unit, Faculty of Tropical Medicine, Mahidol University, Bangkok, Thailand; 3 Wellcome Trust-Mahosot Hospital-Oxford Tropical Medicine Research Collaboration, Microbiology Laboratory, Mahosot Hospital, Vientiane, Lao People's Democratic Republic; 4 Centre for Clinical Vaccinology and Tropical Medicine, Nuffield Department of Clinical Medicine, University of Oxford, Oxford, United Kingdom; 5 Menzies School of Health Research and Northern Territory Clinical School, Royal Darwin Hospital, Darwin, Northern Territory, Australia; 6 Microbiology and Immunology, James Cook University, Townsville, Australia; 7 Center for Microbial Genetics and Genomics, Northern Arizona University, Flagstaff, Arizona, United States of America; 8 Department of Biological Sciences, Northern Arizona University, Flagstaff, Arizona, United States of America; 9 Oxford University Clinical Research Unit, Hanoi, Vietnam; 10 DSO National Laboratories, Singapore, Singapore; 11 Department of Microbiology, Kasturba Medical College, Manipal University, Manipal, Karnataka, India; 12 Research and Evaluation Department, Duke-NUS Graduate Medical School Singapore, Singapore; 13 Friedrich Loeffler Institute of Medical Microbiology, Ernst Moritz Arndt University of Greifswald, Greifswald, Germany; 14 Department of Medicine, Cambridge University, Addenbrooke's Hospital, Cambridge, United Kingdom; 15 Department of Microbiology and Immunology, Faculty of Tropical Medicine, Mahidol University, Bangkok, Thailand; Oxford University Clinical Research Unit, Viet Nam

## Abstract

**Background:**

*Burkholderia pseudomallei*, a Tier 1 Select Agent and the cause of melioidosis, is a Gram-negative bacillus present in the environment in many tropical countries. Defining the global pattern of *B. pseudomallei* distribution underpins efforts to prevent infection, and is dependent upon robust environmental sampling methodology. Our objective was to review the literature on the detection of environmental *B. pseudomallei*, update the risk map for melioidosis, and propose international consensus guidelines for soil sampling.

**Methods/Principal Findings:**

An international working party (Detection of Environmental *Burkholderia pseudomallei* Working Party (DEBWorP)) was formed during the VIth World Melioidosis Congress in 2010. PubMed (January 1912 to December 2011) was searched using the following MeSH terms: *pseudomallei* or melioidosis. Bibliographies were hand-searched for secondary references. The reported geographical distribution of *B. pseudomallei* in the environment was mapped and categorized as definite, probable, or possible. The methodology used for detecting environmental *B. pseudomallei* was extracted and collated. We found that global coverage was patchy, with a lack of studies in many areas where melioidosis is suspected to occur. The sampling strategies and bacterial identification methods used were highly variable, and not all were robust. We developed consensus guidelines with the goals of reducing the probability of false-negative results, and the provision of affordable and ‘low-tech’ methodology that is applicable in both developed and developing countries.

**Conclusions/Significance:**

The proposed consensus guidelines provide the basis for the development of an accurate and comprehensive global map of environmental *B. pseudomallei*.

## Author Summary

Melioidosis is a serious infectious disease caused by the Tier 1 selected agent and Gram-negative environmental saprophyte, *Burkholderia pseudomallei*. The organism is commonly found in soil and water in melioidosis endemic areas. Infection in humans occurs following bacterial inoculation, inhalation or ingestion. There is a striking lack of accurate information on the global risk of melioidosis, something that could be determined from the global distribution of environmental *B. pseudomallei*. Soil sampling to detect the presence of *B. pseudomallei* has been *ad hoc*, poorly standardized, and the available information poorly collated. Negative studies are almost never reported, and there is no published review on this topic. We responded to this problem during the VI^th^ World Melioidosis Congress held in Townsville, Australia in December 2010 by forming the ‘Detection of Environmental *Burkholderia pseudomallei* Working Party (DEBWorP)’. We have since worked together to undertake a systematic review, map the available information, and reach a consensus on low cost methods for the detection of environmental *B. pseudomallei*. Our goal is to promote the use of these consensus methods and encourage people worldwide to participate in an effort to produce a comprehensive global map of environmental *B. pseudomallei*.

## Introduction

Melioidosis, a community-acquired infectious disease caused by the environmental Gram-negative bacillus *Burkholderia pseudomallei*, was first described in Burma in 1912 [1]. To date, most cases have been reported from northeast Thailand where it is the third most common cause of death due to infectious diseases after HIV/AIDS and tuberculosis [2], and from Darwin in northern Australia where it has been the commonest cause of fatal community-acquired bacteremic pneumonia [3]. Melioidosis is also being increasingly reported from many countries across south and east Asia as well as parts of South America, Papua New Guinea and the Caribbean. It is apparently rare in Africa [4], although infection may pass unrecognized because diagnostic confirmation relies on microbiological culture, which is often unavailable in resource-restricted regions of the world. Even with such facilities, *B. pseudomallei* may be dismissed as a culture contaminant [5], or misidentified by standard identification methods including API20NE and automated bacterial identification systems [6], [7].

Humans acquire melioidosis following contact with *B. pseudomallei* in the environment. A number of epidemiological and animal studies have indicated that melioidosis is not contagious, and that disease is acquired following skin inoculation, inhalation or ingestion of *B. pseudomallei* [8]. Defining the global distribution of environmental *B. pseudomallei* is important for the development of a risk map for melioidosis, since this provides the geographical setting for preventive measures as well as raising awareness of this disease among healthcare workers in affected areas. Environmental sampling can be used to identify areas where people are at risk even before cases are recognized. For example, the first environmental survey around Vientiane City (the capital of Lao PDR) in 1998 demonstrated the presence of *B. pseudomallei* prior to the recognition of human disease [9]. This drove an effort to identify *B. pseudomallei* from clinical specimens, with the first case of melioidosis being identified in 1999 [10], which has been followed by the identification of more than 560 culture-positive melioidosis patients in the past 12 years.

Environmental surveys have provided evidence for the presence of environmental *B. pseudomallei* in geographically defined regions within numerous countries in southeast Asia, Australia, Papua New Guinea, parts of South America and elsewhere [4]. Although this has provided valuable information, these studies lacked standardization in almost all aspects of study design and conduct. Whilst methodological variability has no effect in the event that the result is positive, poor sampling methods may give rise to false negative results and inappropriate reassurances regarding the absence of risk [11]. The information generated to date has also been piecemeal and provides a very incomplete global risk map, with vast regions of the world completely unmapped, including Indonesia, India, Africa, North America and most of South America. In addition, questions extending beyond risk, such as *B. pseudomallei* persistence and bacterial load in soil over time, during different weather conditions and in neighboring regions of the same or adjacent countries cannot be addressed unless the methodology is standardized. Ideally, the sampling technique should be relatively simple and detection of *B. pseudomallei* performed at low cost across the world. However, no protocol or standard operating procedure (SOP) is currently available for investigators to download and use.

Recognising these problems, our objectives were to form a working party of individuals with experience in the detection of environmental *B. pseudomallei*, to use this body to develop consensus guidelines on sampling study design and conduct, to make these freely available to the scientific community, and to facilitate their uptake worldwide by ensuring affordability and simplicity of methodology.

## Methods

### Literature Review

#### Search strategy and study selection

PubMed (January 1912 to January 2011) was searched using the following MeSH terms: melioidosis and *pseudomallei*. The search was limited to studies published in English and French. The predetermined eligibility criterion for inclusion was a study conducted to detect *B. pseudomallei* in the environment. Titles and abstracts were screened for relevance, and bibliographies from selected studies hand-searched for secondary references. Database searching was performed and selected by DL and reviewed by DABD and SJP.

#### Data extraction

A data extraction form to record the methodology used to detect environmental *B. pseudomallei* and study findings was developed and piloted with a subset of the first 20 eligible studies prior to development of a final version (Text S1). In brief, the data extracted related to geographical location, study design, type of sample taken (soil or water), depth of sampling (for soil sampling), amount of soil (in gram) or water (in ml) collected, number of samples collected, the proportion of positive samples, and the methods used to detect and identify *B. pseudomallei*. Data from all studies included in the final review were extracted by DL, reviewed by DABD and SJP, and any disagreement resolved by discussion.

#### Definitions

The presence of environmental *B. pseudomallei* in each country was categorized as being (i) definite, (ii) probable, or (iii) possible (Table 1). ‘Definite’ was defined by the detection of *B. pseudomallei* from the environment using culture or a specific PCR for *B. pseudomallei* with or without evidence of melioidosis having been acquired in that country. ‘Probable’ was defined when no reports were identified in the published literature of environmental sampling but clinical reports indicated in-country disease acquisition. This drew on data from the most recent reviews of the distribution of human melioidosis [4], [12]. ‘Possible’ was defined as the detection of *B. pseudomallei* from the environment using culture or PCR methodology that did not include a confirmatory test for *B. pseudomallei* in a setting that lacked evidence of melioidosis having been acquired in that area/country. This included several countries where the detection of environmental *B. pseudomallei* was reported prior to the description of the highly related *Burkholderia thailandensis* as a separate species in 1998 [13]–[21]. Prior to this, *B. thailandensis* was referred to as ‘non-pathogenic’ or ‘arabinose-positive’ *B. pseudomallei* [22]. *B. pseudomallei* and *B. thailandensis* are indistinguishable on the basis of colony morphology, antimicrobial susceptibility pattern and many biochemical tests (arabinose assimilation being an important exception) [22], [23]. A few early studies inoculated suspected *B. pseudomallei* colonies or environmental samples into an animal model to isolate the organism or determine virulence. This would be predicted to distinguish between *B. pseudomallei* and non-virulent *Burkholderia* spp. [22], and was accepted as ‘definite’ evidence of *B. pseudomallei*. The global map showing the distribution of *B. pseudomallei* was generated by ArcGIS (10.0, Redlands, CA)

**Table 1:** Global distribution of environmental *B. pseudomallei*.

Level of evidence	Definition	Countries
Definite	(1) Organism isolated from soil or water with adequate identification by culture or a *B. pseudomallei*-specific PCR, and (2) Evidence for melioidosis having been acquired in that country	Asia (Cambodia [98], China [31], [48], [49], Iran [50], Lao PDR [9], [63], Malaysia [28], [58], [68], [77], Singapore [78], [99], Sri Lanka [51], Taiwan [52]–[55], Thailand [11], [15], [16], [37], [39], [57], [60], [64]–[66], [84], [85], [100], [101] and Vietnam [19], [26], [102], [103]), Oceania (Australia, [17], [18], [20], [24], [25], [27], [38], [42], [59], [61], [62], [71]–[75], [104] and Papua New Guinea [67]), Africa (Burkina Faso [40], Madagascar,[14], Niger [40]), Europe (France [14], [29], [30])*, and, South America (Brazil [14], [46], [47])
Probable	(1) No report identified of *B. pseudomallei* isolation from soil or water, and (2) Evidence for melioidosis having been acquired in that country	Asia (Bangladesh, Brunei, Egypt, India, Indonesia, Myanmar, Pakistan, Philippines and Saudi Arabia), Ocenia (Fiji), Africa (Chad, Gambia, Kenya, Nigeria, Sierra Leone, South Africa and Uganda), Central America (Costa Rica, El Salvador, Honduras, Mexico and Panama), South America (Colombia, Ecuador, Puerto Rico and Venezuela), Europe (Turkey), and Others (Aruba, Guadeloupe, Guam, Mauritius, Martinique, New Caledonia, Puerto Rico) [4], [12]
Possible	(1) Organism isolated from soil or water that was considered to be *B. pseudomallei*, but (2) identification process not sufficient to exclude other, non-pathogenic environmental *Burkholderia spp*. such as *B. thailandensis*, and 2) No evidence for melioidosis having been acquired in that country	Côte d'Ivoire [14], Haiti [14], Italy [21] and Peru [14]

* In France, soil culture positive for *B. pseudomallei* was initially reported in the ‘Jardin des Plantes’ in Paris after an outbreak of animal melioidosis which was thought to have originated from a panda imported from China, and the organism was subsequently reported to have been detected in soil throughout the country [14], [29], [30]. There is no evidence to suggest its continuing presence.

### Recommendations

#### Forming the working party

The Detection of Environmental *Burkholderia pseudomallei* Working Party (DEBWorP) was formed during the VI^th^ World Melioidosis Congress held in Townsville, Australia in December 2010. Following an announcement of the initiative, interested individuals were identified, the consortium formed, and email used to communicate with its members.

#### Development of consensus on the detection of *B. pseudomallei* in soil

A questionnaire was formulated by four investigators (DL, DABD, BC and SJP) based on areas of variation in practice relating to study design and methodology for the detection of *B. pseudomallei* in soil (Text S2). This was sent to all members of DEBWorP. Answers and comments were collated, and areas of common and variant practice identified. A second questionnaire was developed to cover areas of variant practice, which was again sent to all members. Recommendations on best practice were reached based on a combination of information from both questionnaires, and circulated to the working party members for final approval. The recommendations did not include study design and methodology for detection of *B. pseudomallei* in air or water, or quantitation of *B. pseudomallei* in soil.

## Results and Discussion

### Literature Review

**Figure 1:**
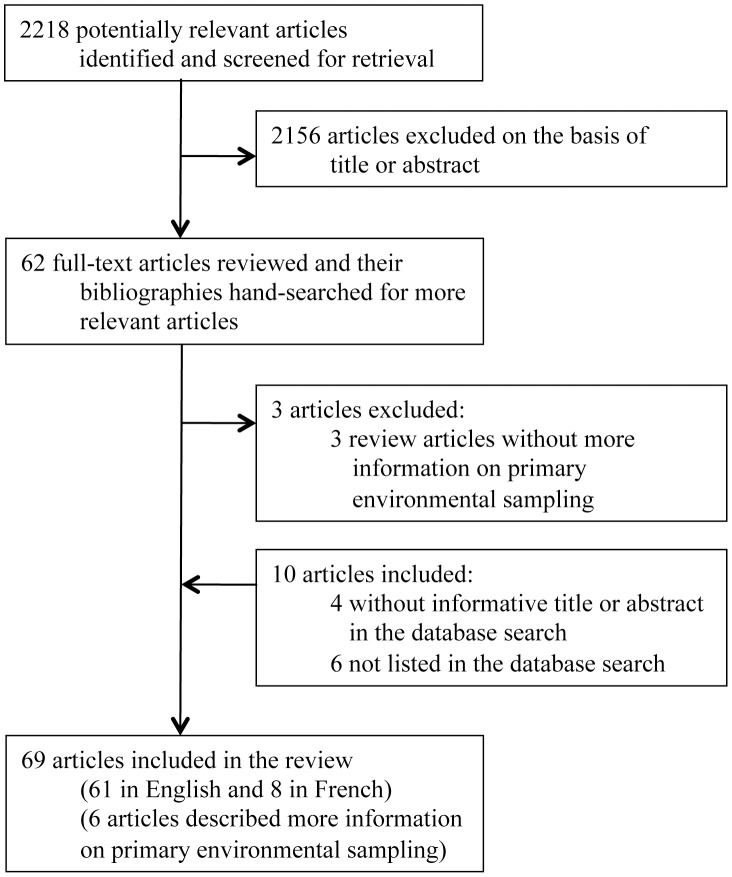
Flow diagram showing study selection.

The search terms used identified 2,218 articles, 62 of which remained after screening of titles and abstracts (Figure 1). These were retrieved and the full text reviewed. An additional 10 articles were identified from the bibliography of the 62 articles which had been missed during the primary search either because they did not have an informative title or abstract (n = 4) [14], [19], [24], [25], or were not listed on PubMed (n = 6) [26]–[31]. Three review articles without additional information on primary environmental sampling were excluded [32]–[34]. Eight articles described more information on previous environmental sampling studies and were included [11], [29], [30], [35]–[39]. Therefore, 69 articles reporting 61 environmental studies for the presence of *B. pseudomallei* published between 1912 and 2011 were included in the review (Table 1 and Table S1).

A total of 50/61 (82%) environmental studies reported the detection of environmental *B. pseudomallei* identified using culture and/or a PCR specific for *B. pseudomallei* (Table S1). Strains collected in France [14], Burkina Faso [40], Madagascar [14], and Niger [40] were later confirmed as *B. pseudomallei* by genotyping [41]. Another 7/61 studies reported the detection of environmental *B. pseudomallei* using culture and/or PCR, but did not exclude the possibility that isolates were other, non-pathogenic environmental *Burkholderia spp*. [13], [15]–[18], [20], [21], [38]. Only 3/61 studies (one from Kenya and two from Australia) reported negative environmental surveys for *B. pseudomallei* [25], [42], [43], and a study from the USA in 1977 identified a *B. pseudomallei-like* organism which was later identified as *B. oklahomensis* [44], [45].

#### Global distribution of environmental *B. pseudomallei*

**Figure 2:**
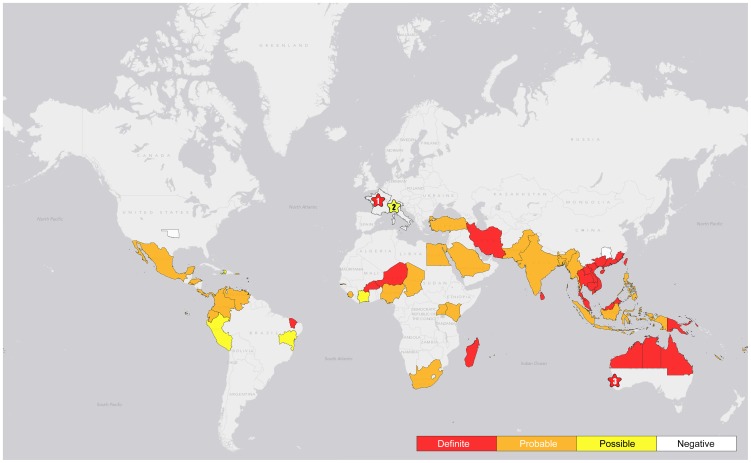
Global map showing the distribution of *B. pseudomallei*. Definitions of definite, probable and possible presence of environmental *B. pseudomallei* are described in Table 1. ^1^ represents ‘Jardin des Plantes’ in Paris where soil cultures positive for *B. pseudomallei* were initially reported after an outbreak of melioidosis, which was thought to have originated from a panda imported from China [14]. ^2^ represents Bologna, Italy, where *B. pseudomallei* in tap water (6 out of 85 specimens) was reported in 2000 [21]. However, confirmation of *B. pseudomallei* by specific identification methods was not reported. ^3^ represents Chittering, southwest Western Australia, where *B. pseudomallei* was isolated and confirmed from a single soil specimen in 1980, following the outbreak of melioidosis in animals [17], [38]. There has been no evidence of environmental *B. pseudomallei* in southwest Western Australia since then.

There was ‘definite’ evidence for the presence of environmental *B. pseudomallei* in 17 countries (Table 1 & Figure 2). Eight were either in southeast Asia (Cambodia, Lao PDR, Malaysia, Singapore, Thailand and Vietnam) or Oceania (Australia and Papua New Guinea), with the remainder (n = 9) being Brazil [46], [47], Burkina Faso [40], China [31], [48], [49], France [14], Iran [50], Madagascar [14], Niger [40], Sri Lanka [51] and Taiwan [52]–[55]. The area sampled within each country was nearly always limited (Table S1). In France, soil culture positive for *B. pseudomallei* were initially reported in the ‘Jardin des Plantes’ in Paris after an outbreak of animal melioidosis, which was thought to have originated from a panda imported from China, but the organism was subsequently reported to have been detected in soil throughout the country [14], [29], [30]. Although one clinical and one environmental strain isolated in France were later confirmed as *B. pseudomallei* by genotyping [41], there was insufficient information given about the identification of *B. pseudomallei* isolated from multiple soil samples collected from across France to be entirely sure of their identity, and they are not available for further testing [14], [29], [30]. Importation followed by environmental treatment to eradicate *B. pseudomallei* will result in a change in classification, but it is unclear from the literature whether *B. pseudomallei* has been eradicated in France. A further 34 countries were assigned to the ‘probable’ category based on clinical evidence of indigenous melioidosis but lack of environmental studies. Two studies described the molecular identification or genotyping of environmental *B. pseudomallei* isolates from Ecuador, Kenya and Venezuela [41], [56], but no environmental sampling studies positive for *B. pseudomallei* were identified for these countries in the published literature. A total of 4 countries including Côte d'Ivoire [14], Haiti [14], Italy [21] and Peru [14] were assigned to the ‘possible’ category (Table 1 & Figure 2) based on inadequate bacterial confirmation of putative environmental *B. pseudomallei* combined with a lack of evidence for indigenous melioidosis.

#### Sampling strategies used for the detection of environmental *B. pseudomallei*

Published sampling strategies for the detection of environmental *B. pseudomallei* are shown in Table 2. Sampling was performed in both dry and wet seasons, and sampling duration ranged from 1 day to 3 years [57]. A consistent difference in positivity rates between the wet and dry season was not established. Three studies found a higher positivity rate in the wet season [35], [58], [59], and two studies reported a higher positivity rate in the dry season [20], [60]. A recent study found that, in a given region, most areas had higher positivity in the wet season but some had a higher positivity in the dry season, which suggested that other factors such as the presence of animals or land use also contribute to differences in positivity rates between wet and dry seasons [61].

**Table 2:** Published and recommended sampling strategies for the isolation of *B. pseudomallei* from soil.

Sampling strategy	Published sampling strategy	Consensus guideline
Sample size calculation	Not stated and often low sample size	Sample size calculation should be presented and should correspond with the aims of the study
Sampling site selection	Variable, including random site selection and practical considerations (e.g. sampling at points along a main road)	For pilot studies that are conducted to identify environmental *B. pseudomallei* in areas where sampling has not been done previously, choose sites most likely to be positive based on available information such as areas around households or working fields of melioidosis patients. If such information is unavailable, use the GIS program to randomly select sampling sites
		For large environmental surveys in areas where *B. pseudomallei* is known to be present in the environment, use the GIS program to randomly select sites across the designated region
Sampling points per site	Ranged from 2 to 100 points per field	Use a fixed interval sampling grid
		To determine presence of *B. pseudomallei* in one field (around 50×50 sq meters), 100 points per site
		To determine presence or distribution of *B. pseudomallei* in a wider area, number of points per site and number of sites should be calculated based on geo-statistical sample size calculation which should provide the confidence level required
Distance between sampling point within a sampling site	1 to 5 meters, or not reported	If no prior information available for *B. pseudomallei* distribution in test area, take samples at a distance of 2.5 to 5 meters apart
		If prior information is available, samples should be taken at an optimal distance based on geo-statistical sample size calculation
Soil sampling depth	Ranged from 0 to 90 cm of depth	30 cm depth
Weight of soil sample per sampling point	Ranged from 2 to 1,000 gram of soil	10 gram of soil (put into universal tube)
Temperature during transportation of sample to laboratory	Variable, including room temperature and refrigerated temperature	At ambient temperature and away from direct sunlight or heat source
		Process soil samples as soon as possible

Of 61 studies, 55 evaluated the presence of *B. pseudomallei* in soil, and 35 in water. The majority of studies chose sampling sites on an *ad hoc* basis. Of 54 studies with information about land use for the sampling site, 20 were conducted in rice fields and 35 in other areas including animal pens, residential areas around the homes of cases, forests, scrubland, and agricultural fields containing other crops. Most studies collected a low number of samples (2 to 7) per study site, and did not provide a detailed description of the sampling design or strategy, sample size calculation or distance between sampling points within each site. Three articles described random selection of the study site in a given area using GPS, and provided a detailed sampling strategy [61]–[63]. The largest number of samples collected from a single site was 100, in which samples were collected using a fixed interval grid [63]–[65]. Soil sampling depth ranged from surface to 90 cm. The weight of each soil sample collected ranged from 2 to 1,000 grams [18], [43].

#### Methods of *B. pseudomallei* detection in soil

The methodology used to detect *B. pseudomallei* in soil samples has 2 main stages: (i) bacterial extraction, and (ii) detection methods using culture or PCR (or historically, animal inoculation) (Table 3). The process of bacterial extraction involves the addition of a solution to the soil, mixing with various degrees of homogenization, and a period of settling prior to removal of the supernatant. The solution used has varied between distilled water, normal saline, detergent solution [66], or enrichment media, with a variable soil to solution ratio (wt/vol) ranging from 2∶1 to 1∶10 [49], [57]. The method used to mix the soil and solution has varied between manual shaking, vortexing or use of an orbital shaker. The time period used to mix the solution has varied from less than 1 minute to 48 hours [61], [67], and the time for soil sedimentation after mixing from 5 minutes to 24 hours [66], [67]. The volume of fluid used for culture has varied from 0.5 to 10 ml of supernatant [60], [67], or the spun deposit of 80 ml of supernatant [35]. The volume used for DNA extraction prior to PCR has varied from 3 ml of supernatant [53], the deposit of 20 ml of supernatant [61], [62], or direct extraction from different weight of soil [39], [52], [54]. The volume used for guinea-pig or hamster inoculation has varied from 1 to 2 ml [57], [68].

**Table 3:** Published and recommended methodologies for the isolation of *B. pseudomallei* from soil.

Methodologies	Published methods	Consensus guideline
*B. pseudomallei* extraction solution	Distilled water, normal saline, detergents or enrichment media	Threonine-basal salt plus colistin 50 mg/L (TBSS-C50 broth)
		Ashdown broth containing colistin and crystal violet is an alternative
Ratio of soil and extraction solution (wt/wt)	Ranged from 2∶1 to 1∶10	1∶1 (10 gram of soil to 10 ml of TBSS-C50 or Ashdown broth)
Extraction method	Manual shaking, vortexing or orbital shaker	Vortexing for 30 seconds
		Manual mixing of soil is an alternative option, and may be required if sample is compacted
Techniques for detection of *B. pseudomallei*	Culture, PCR or animal inoculation	Culture (PCR could be added as an additional technique if available)
Protocol for culture	Variable, including direct culture on solid media and quantitation, and qualitative methods relying on broth enrichment	Incubate the specimen (universal tube with 10 gram of soil plus 10 ml TBSS-C50 or Ashdown broth) for 48 hours
Temperature of incubator	Variable, ranged from 37 to 42°C	40°C is recommended, and 37–42°C is an alternative option
Protocol for sub-culture	Variable	Subculture 10 µL of supernatant onto an Ashdown agar plate, and streak to achieve single colonies
		Incubate plate and examine every 24 hours for 7 days
Identification of *B. pseudomallei*	Variable, including basic microbiological tests (which include typical colony morphology, Gram stain, positive oxidase test, inability to assimilate arabinose, resistance to gentamicin and colistin with susceptibility to co-amoxiclav) and biochemical kits (including API20NE [105] and Vitek) with or without additional confirmatory tests (specific latex agglutination test [89], or a specific PCR assay [62], [75], [91], [93])	Basic microbiological tests (which include typical colony morphology, Gram stain, positive oxidase test, inability to assimilate arabinose, resistance to gentamicin and colistin with susceptibility to co-amoxiclav) is mandatory plus at least one confirmatory test (API20NE, Vitek system, specific latex agglutination test [89] or a specific PCR assay [62], [75], [91], [93], unless latex test or PCR assay was used during screening)
		Specific latex agglutination test [89], or a specific PCR assay [62], [75], [91], [93] can be used a screening test

The most common detection method has been culture using selective media (n = 46). Most protocols used a selective enrichment broth (n = 44), with a variable specimen to medium ratio (vol/vol) ranging from 1∶1 to 1∶20 [67]. The broth used varied and included tryptone soya broth plus crystal violet (5 mg/l) and colistin (20 or 50 mg/l) (CVCB or Ashdown broth) [69], and L-threonine buffered salt solution (TBSS or Galimand and Dodin broth) [14] with or without colistin (20 or 50 mg/l). Culture of bacterial extraction solution on selective agar plates was described in 15 studies, and Ashdown agar was commonly used [69]. The volume of fluid inoculated onto each agar plate varied from 10 to 400 µl [9], [64]. Temperature of incubation varied between 30 and 42°C [19], [67]. The overall efficiency of different techniques at each stage has not been adequately compared. In eight studies using both culture and PCR, the positivity rate for *B. pseudomallei* was higher by PCR than by culture [20], [39], [51]–[55], [62], [64], [70].

#### Methods used to detect *B. pseudomallei* in water

The methodology used to detect *B. pseudomallei* in water samples has 2 main stages: (i) bacterial concentration, and (ii) detection methods using culture or animal inoculation. The volume of each water sample collected ranged from 1 to 5,000 ml [24], [59], [71]–[74]. The method used for bacterial concentration has varied between filtration, centrifugation [43], or precipitation with potassium alum [27]. Filter pore size has varied from 0.20, 0.22 or 0.45 µm [18], [21], [25], [42], [73]–[76]. The volume of fluid used for direct culture was 50 ml, from which the bacteria were extracted either by centrifugation [43], or using potassium alum [27]. The volume used for guinea-pig or hamster inoculation has varied from 1 to 2 ml [24], [58], [68], [71], [77], [78]. The first evidence of *B. pseudomallei* in water came from a study published in 1937 which involved immersion of a guinea pig in water following scarification of its abdomen, following which *B. pseudomallei* was isolated from its blood [26]. The relative sensitivity of detection using culture versus animal inoculation has not been reported.

#### Methods used to detect *B. pseudomallei* in air

There are no studies in PubMED that report air sampling for *B. pseudomallei*. An MSc thesis written by Kinoshita contains details of the culture of *B. pseudomallei* from air at the Hong Kong oceanarium in 1989, 1993 and 1995 [31]. The sampling technique used was to hold an agar plate at about shoulder level to oncoming winds during a typhoon. Kinoshita repeated air sampling by collecting 171 typhoon samples between 1999 and 2002, but all were culture negative for *B. pseudomallei* [31].

### Recommendations on the Detection of *B. pseudomallei* in Soil

All 16 members of DEBWorP agreed that the first recommendations would focus on soil sampling alone, and that there was not enough evidence for recommendations to be made on the detection of *B. pseudomallei* in water and air. All members completed the original version of the questionnaire about variations in study design and methodology for the qualitative detection of *B. pseudomallei* in soil (Text S2). A second iteration was developed after identifying additional issues that could not be resolved without further consultation. All 16 members completed the second version, after which consensus recommendations were developed, sent to all members for comments, and agreed upon. Specific recommendations are shown in Table 2–4, the basis for which is described below.

**Table 4:** Publishing the findings of studies conducted to isolate *B. pseudomallei* from soil.

Reporting the findings	Published findings	Consensus guideline
After publication, deposit raw data to website	Variably reported	After publication, raw data can be deposited to website www.melioidosis.info at the discretion of PI and sponsor of each study
GPS location of study site	Variably reported	After publication, GPS data can be deposited to website www.melioidosis.info at the discretion of PI and sponsor of each study taking account of issues of anonymity.
Positivity rate in each study site and pattern of positivity in each study site	Variably reported	Describe in the manuscript if available.
		After publication, details of results can be deposited to website www.melioidosis.info at the discretion of PI and sponsor of each study
Soil type and history of land use	Variably reported	Describe the current land use in the manuscript, together with the history of land use if available
		Describe the soil texture using previously described method such as ribbon test [97].
		After publication, details of results can be deposited to website www.melioidosis.info at the discretion of PI and sponsor of each study
Sampling time and weather at sampling time point (e.g. rainfall, season)	Variably reported	Describe in the manuscript.
		After publication, details of results can be deposited to website www.melioidosis.info at the discretion of PI and sponsor of each study

#### Choice of sampling site and strategy

The most appropriate sampling strategy will depend on the objectives of the study, and whether any information is already available for the geographical area to be sampled (Table 2). For pilot studies that are conducted to identify *B. pseudomallei* in the environment in areas where sampling has not been performed previously, investigators should gather any available information about possible or definite melioidosis cases in the locality, and sampling site selection should target their residence or work place. In the absence of such information, a less targeted approach will be required in which GIS (geographic information system) software is used to support the random identification of several pilot locations in a given region or country. For large environmental surveys in areas where *B. pseudomallei* is known to be present in the environment, selection of sampling sites using GIS software is also recommended. Within a given location (study site), we recommend the use of a fixed interval grid based on its simplicity and the need for standardization.

#### Number of samples

Taking an insufficient number of soil samples from a designated sampling site runs the risk of a false negative result [11]. This may be due to insensitive detection methods, or because saprophytic bacteria exist in aggregates and can give rise to hot spots and intervening areas that are negative for a specific bacterium. This has been shown to be the case for *B. pseudomallei* [11]. Because of this, random sampling methods using a low sample size may be associated with a low power of detection and a high false negative (type II error) rate [79]. This can be avoided by increasing the number of samples taken [11]. Based on statistical considerations, to determine the presence of *B. pseudomallei* in an area of around 50×50 sq meters, a minimum of 100 sampling points is suggested. This is strongly supported by a recent study in Lao PDR in which one field was deemed positive based on only 1 out of 100 positive sampling points [63].

If a region is already known or highly suspected to be positive for *B. pseudomallei*, an alternative approach is to use adaptive sampling in which a pilot study is performed in a defined experimental area in which a number of random points (e.g. 20) are sampled. If any are positive for *B. pseudomallei*, this confirms the presence of the organism and is sufficient to define this as an area of risk for humans and livestock. If all samples are negative, a second round of sampling is done in which 100 samples are taken from the same site using a fixed interval grid. To determine the presence or distribution of *B. pseudomallei* in a wider area, the number of samples taken per site and the number of sites investigated could be calculated based on a geo-statistical sample size calculation [80], [81].

#### Distance between samples

The presence of hot spots for a specific bacterium in the environment leads to an effect described by the term ‘spatial autocorrelation’, which influences the distance required between each sampling point. What this means in practice is that sampling points adjacent to each other are more likely to yield the same result (e.g. a sample next to a negative sample is likely to be negative) [11]. The distance over which counts of a given environmental bacterium are related (range of spatial autocorrelation) can be defined using a geostatistical tool called the semivariogram [80]. Ideally, the effect of spatial autocorrelation would be factored in to the sampling strategy for *B. pseudomallei*, but this value is likely to be influenced by physicochemical soil parameters and vegetation [82], and vary between and possibly within countries. Therefore, it is not practical to define this prior to formal sampling in most settings. Studies in Thailand suggest that the distance between samples should be between 2.5 and 5 m apart [11], although it is uncertain whether this applies elsewhere. Given the paucity of data on the optimal distance between samples we suggest that sampling be performed 2.5 to 5 m apart, accepting that this is somewhat arbitrary. The optimal sampling distance specific to the study region could be subsequently estimated based on the results of pilot study data for 100 sampling points for one or more sites [80].

#### Soil sampling: quantity, sampling depth and transport to the laboratory

We recommend a depth for soil sampling of 30 cm. This is based on published evidence that the proportion of samples that are culture positive for *B. pseudomallei* is higher at 30 cm than at a shallower depth, but comparable to samples taken deeper than 30 cm [35], [47], [53], [60], [62], [83], [84]. The quantity of soil collected per sample has varied markedly in published studies, and there is no evidence that collecting a greater weight of soil is associated with a higher sensitivity. We suggest taking a weight of 10 grams per sample based on practicality and ease of methodology [85]. As there is evidence showing that survival of *B. pseudomallei* is decreased at low temperatures [86], soil samples should be kept at ambient temperature (24 to 32°C) and away from direct sunlight or heat source during transportation to the laboratory. The specimen should be processed as soon as possible.

#### Extraction of bacteria from soil, and detection and identification of *B. pseudomallei*

We recommend the use of culture as the standard method for environmental *B. pseudomallei* detection in the context of global mapping efforts on the basis of simplicity, specificity and low cost (Table 3). The optimal ratio of soil to extraction solution, mixing technique and sedimentation time are not known. Selective broths have been compared in both laboratory [87] and field settings [20], [60]. We proposed that each 10 gram soil sample be placed into a universal tube, mixed with 10 ml of enrichment medium (either TBSS with colistin 50 mg/l (TBSS-C50) or Ashdown broth), vortexed for 30 seconds, and incubated at 40°C in air for 48 hours. Based on scientific evidence and agreement of the working party, TBSS-C50 is recommended as the primary enrichment medium with Ashdown broth as an alternative. A volume of 10 µl of the upper layer of enrichment medium should be streaked to achieve single colonies onto a whole Ashdown agar plate, incubated at 40°C in air and examined every 24 hours for 7 days. This incubation temperature was chosen based on evidence that it allows growth of *B. pseudomallei* [88], but is inhibitory to some other soil flora (personal observation by DABD and VW). However, incubation at 37°C is acceptable in the event that resources are not available to incubate at 40°C. Subculture of 10 µl is based on experience in Thailand and represents a balance between detection of *B. pseudomallei* and limiting the bioburden of other flora that grow on the agar plate. Subculture of higher volumes (100 µl) may be associated with a higher yield although there currently is no published evidence to support this.

Several steps of the method recommended here (direct culture of 10 gram of soil in 10 ml of TBSS-C50 and subculture onto Ashdown agar) are based on methods in widespread use in Australia [62], [75]. Furthermore, the sensitivity of our recommended method was recently compared to a more laborious method which has been used extensively in Thailand [85]. The latter involves collection of 100 gram of soil which is mixed with 100 ml of distilled water, left to settle overnight, and the upper layer of water removed for culture on Ashdown agar and in TBSS-C50. In the comparative study, 94 out of 200 soil samples were culture positive for *B. pseudomallei* [85]. Yield was not different between the two methods (70/94 vs. 79/94 respectively; p = 0.15), supporting the use of our currently recommended method.

#### Identification of *B. pseudomallei*

Any colony with a colony morphology suggestive of *B. pseudomallei* can be tested by basic microbiological tests (typical colony morphology on Ashdown agar, Gram stain, positive oxidase test, inability to assimilate arabinose, resistant to gentamicin and colistin, susceptible to co-amoxiclav) followed by confirmatory tests (specific latex agglutination test [89], a specific PCR assay [53], [55], [62], [75], [90]–[95], or validated identification kits such as API20NE or Vitek system). The API20NE database does not include a profile of *B. thailandensis*, which give results that are similar to those for *B. pseudomallei* except that *B. thailandensis* is positive for arabinose assimilation. For rapid evaluation, a specific latex agglutination [89], [96] or PCR assay [53], [55], [62], [75], [90]–[95] could be used as a screening test, followed by basic microbiological tests to complete the identification process.

#### Data presentation and data sharing

We propose that publication of studies on environmental detection of *B. pseudomallei* include the positivity rate and pattern of positivity over 100 sampling points, history of land use, date of sampling, weather conditions and soil texture (%sand, loam and clay) using the methodology described previously [97] (Table 4). DEBworP is in the process of developing a website (www.melioidosis.info) where complete data from sampling studies can be deposited with the assistance of a curator (DL), and at the discretion of the principal investigator and sponsor of each study. This will be used to build an interactive global map of the distribution of environmental *B. pseudomallei*, as well as those places where melioidosis has been acquired in humans and animals. The website will also provide downloadable protocols describing methodology for soil sampling and culture, including details of each reagent and test used (Text S3). The recommended protocols have been successfully used in Thailand [85], although further evaluation of these is required in different countries.

Although the methodology presented here aims to reduce the risk of false negative sampling surveys, this is unlikely to be perfect. As a result, a single negative sampling survey does not represent definite evidence that the site is free of *B. pseudomallei*, although it would be predicted to reflect a region of much lower risk compared with a positive site. The need to undertake further sampling requires consideration of risk-benefit. There is also considerable scope to improve on the methodology described here, including improvement in the sensitivity of culture which could include the development of media that are even more selective for *B. pseudomallei* in soil, and ultimately the development of easy-to-use and accurate diagnostic kits for environmental sampling. Our recommendations will be updated in the future as and when new information or knowledge becomes available.

#### Concluding comments

Our knowledge of the global distribution of *B. pseudomallei* is incomplete, and the methodology to determine the presence of this organism in the environment has not been standardized and is liable to false negativity (if insufficient samples are taken or inappropriate techniques are used), and false positivity (if methods are not adequate to exclude related *Burkholderia* species). We have provided consensus guidelines on strategies and methodologies to determine the presence of *B. pseudomallei* in soil that are simple and applicable in settings with limited resources. To develop a complete risk map of melioidosis, our working party aims to support and promote environmental studies on a global scale, supported by a website (www.melioidosis.info) with downloadable protocols and a mechanism for data collection and sharing.

## Supporting Information

Table S1 Characteristics of studies included in the review.
(DOC)

Table S2 PRISMA checklist.
(DOC)

Text S1 Data extraction form for studies that determined the presence of *Burkholderia pseudomallei* in the environment.
(DOC)

Text S2 Questionnaire on the detection of environmental *Burkholderia pseudomallei*.
(DOC)

Text S3 Standard Operating Procedure (SOP): simplified method for the isolation of *Burkholderia pseudomallei* from soil.
(DOC)
