# Supplementary material for: Systematic Review and Consensus Guidelines for Environmental Sampling of Burkholderia pseudomallei
Source: PLoS Negl Trop Dis. 2013 Mar 21;7(3):e2105. doi: 10.1371/journal.pntd.0002105 (PMC3605150; doi:10.1371/journal.pntd.0002105)
Supplement: Table S1 — Characteristics of studies included in the review. (DOC) [file pntd.0002105.s001.doc]

**Table S1.** Characteristics of studies included in the review

| Publications [Reference] | Country | Region | *B. pseudomallei* detection method | Soil samples  No. collected/No. positive (%) | Water samples  No. collected/No. positive (%) | | *B. thailandensis* exclusion method* |
| --- | --- | --- | --- | --- | --- | --- | --- |
| Vaucel (1937) | Vietnam | Hanoi | Guinea-pigs | Not Done | 1 / 13 (8%) | | Virulence test |
| Chambon (1955) | Vietnam | Saigon | Guinea-pigs | 1 / 80 (1%) | 4 / 70 (6%) | | Virulence test |
| Lerclerc and Sureau  (1956) | Vietnam | Hanoi | Bacteriophage | Not Done | 30 / 152 (20%) | | Not done |
| Laws and Hall (1964) | Vietnam | Hanoi | Culture and Guinea-pigs | 0 / 6 (0%) by culture  0 / 6 (0%) by Guinea-pigs | 0 / 48 (0%) by culture  2 / 48 (4%) by Guinea-pigs | | Virulence test |
| Strauss et al (1967) | Malaysia | Sabah | Hamsters | 1 / 57 (2%) | 11 / 354 (3%) | | Virulence test |
| Ellison et al (1969) | Malaysia | Kuala Lumpur | Hamsters | 20 / 1078 (2%) | 32 / 1120 (3%) | | Virulence test |
| Strauss et al (1969) | Malaysia | East states | Hamsters | 11 / 287 (4%) | 425 / 5621 (8%) | | Virulence test |
| Strauss et al (1969) | Malaysia | Selangor | Hamsters | 1 / 228 (0.4%) | 88 / 2229 (4%) | | Virulence test |
| Thin (1971) | Singapore | Singapore | Hamsters | Not done | 8 / 136 (6%) | | Virulence test |
| Dodin and Ferry (1974) | Burkina Faso, Niger | Not stated | Culture | 6 / 8 (75%) | 11 / 31 (35%) | | Virulence test and later by MLST |
| Pourtaghva et al (1975) | Iran | Teharan | Culture | 19 / 157 (12%) | Not done | | Virulence test |
| McCormick et al (1977) | USA | Oklahoma | Culture | 1 sample positive | Not done | | Later identified as *B. oklahomensis* |
| Thomas et al (1977) | Australia | Townsville | Culture | 9 / 730 (1%) | 3 / 30 (10%) | | Virulence test |
| Ashdown (1979) | Australia | Townsville | Culture | 1 / 20 (4%) | Not done | | Virulence test |
| Thomas et al (1981) | Australia | Townsville | Culture | 1 / 19 (5%) | 0 / 15 (0%) | | Virulence test |
| Galimand and Dodin (1982)  . | Brazil, Cote d’Ivoire,  France, Haiti,  Hong Kong,  Madagasgar, Peru | Not stated | Culture | Not described | | | Later by MLST for the strains from France and Madagasgar |
| Achana et al (1985) | Thailand | Songkla | Culture | 1 sample positive | | 1 sample positive | Not done |
| Nachiangmai et al (1985) | Thailand | Songkla | Culture | 81 / 169 (18%) | | 6 / 118 (5%) | Not done |
| Ketterer et al (1986) | Australia | Queensland | Culture | 0 / 25 (0%) | | 0 / 39 (0%) | Not applicable |
| Golledge et al (1992) | Australia | Western Australia | Culture | Unidentified number of samples positive | | Not Done | Not done |
| Merianos et al (1993) | Australia | Northern Territory | Culture | 3 / 68 (4%) | | 1 / 18 (6%) | Not done |
| Van Phung et al (1993) | Vietnam | Bac Hong and Duong Lam | Culture | 4 / 240 (2%) | | 1 / 190 (1%) | API20NE |
| Batchelor et al (1994) | Kenya | Kilifi | Culture | 0 / 81 (0%) | | 0 / 81 (0%) | Not applicable |
| Smith et al (1995) | Thailand | Ubon Ratchathani | Culture | 11 / 168 (7%) | | Not done | Arabinose test |
| Wuthiekanun et al (1995) | Thailand | Ubon Ratchathani | Culture | 130 / 1075 (12%) | | Not done | Arabinose test |
| Yang et al (1995) | China | Hainan, Fujian, Guangxi and Guangdong | Culture | 58 / 1366 (4%) | | Not done | Virulence test |
| Yap et al (1995) | Singapore | Singapore | Hamsters | 3 samples positive | | Not done | Virulence test |
| Brook et al (1997) | Australia | Darwin | Culture and PCR | 14 / 72 (19%) by culture  19 / 52 (37%) by PCR | | Not done | Not done |
| Parry et al (1999) | Vietnam | Ho Chi Minh City | Culture | 15 / 407 (4%) | | Not done | Arabinose test |
| Vuddhakul et al (1999) | Thailand | North, Northeast, Central and South | Culture | 232 / 3585 (6%) | | Not done | Arabinose test |
| Finkelstein et al (2000) | Thailand | Northeast and South | Hamsters | 151 / 1300 (5%) | | Not done | Virulence test |
| Inglis et al (2000) | Australia | Western Australia | Culture | 1 sample positive | | 6 samples positive | PCR |
| Zanetti et al (2000) | Italy | Bologna | Culture | Not done | | 6 / 85 (7%) | Not done |
| Currie et al (2001) | Australia | Northern Territory | Culture | 7 / 87 (8%) | | 1 / 35 (3%) | Arabinose test |
| Kao et al (2003) | Taiwan | Fan-Sang | Culture and PCR | 66 / 900 (7%) by culture  79 / 900 (8%) by PCR | | Not done | PCR |
| Kinoshita (2003) | China | Hong Kong | Culture | 6 / 429 (1%) | | 6 / 110 (5%) | API20NE, latex agglutination test |
| Inglis et al (2004) | Australia | Darwin | Culture | 11 / 360 (3%) | | 11 / 385 (3%) | In-house agglutination test and PCR |
| Rolim et al (2005) | Brazil | Ceara | Culture | 1 sample positive | | 1 sample positive | In-house agglutination test and PCR |
| Wuthiekanun et al (2005) | Lao PDR | Vientiane | Culture | 40 / 110 (37%) | | Not done | Latex agglutination test |
| Kaestli et al (2007) | Australia | Darwin | Culture and PCR | 13 / 104 (13%) by culture  20 / 104 (19%) by PCR | | Not done | PCR |
| Su et al (2007) | Taiwan | Southwestern | Culture and PCR | 24 / 311 (8%) by culture  34 / 311 (11%) by PCR | | Not done | PCR |
| U'ren et al (2007) | Thailand | Khon Kaen | Culture | 68 / 450 (15%) | | Not done | PCR |
| Chantratita et al (2008) | Thailand | Ubon Ratchathani | Culture | 80 / 100 (80%) | | Not done | Latex agglutination test |
| Inglis et al (2008) | Sri Lanka | Peradeniya | Culture and  PCR | No samples culture positive  Unidentified number of samples PCR positive | | Not done | PCR |
| Levy et al  (2008) | Australia | Western Australia | Culture | No *B. pseudomallei* isolated | | No *B. pseudomallei* isolated | Not applicable |
| Palasatien et al (2008) | Thailand | Khon Kaen | Culture | Unidentified number of samples positive / 344 | | Not done | Arabinose test and PCR |
| Warner et al (2008) | Papua New Guinea | Western region | Culture | 7 / 274 (3%) | | Not done | Arabinose test and PCR |
| Wuthiekanun et al (2008) | Cambodia | Siem Reap | Culture | 12 / 40 (30%) | | Not done | Arabinose test |
| Inglis et al (2009) | Australia | Kimberley | Culture and PCR | 6 samples positive by culture  15 samples positive by PCR | | 6 samples positive by culture  15 samples positive by PCR | PCR |
| Kaestli et al (2009) | Australia | Darwin | PCR | 107 / 809 (13%) | | Not done | PCR |
| Rolim et al (2009) | Brazil | Ceara | Culture | 26 / 600 (4%) | | Not done | In-house agglutination test and PCR |
| Wuthiekanun et al (2009) | Thailand | Ubon Ratchathani | Culture | 28 / 100 (28%) | | Not done | Latex agglutination test |
| Chen et al (2010) | Taiwan | Taiwan | Culture and PCR | 62 / 1053 (6%) by culture  115 / 1053 (11%) by PCR | | Not done | 16S RNA |
| Draper et al (2010) | Australia | Darwin | Culture | Not done | | 16 / 47 (34%) | PCR |
| Ma et al (2010) | China | Guangxi | Culture | 13 / 154 (8%) | | 0 / 130 (0%) | 16S RNA |
| Baker et al (2011) | Australia | Townsville | PCR | 33 / 80 (41%) | | 51 / 56 (91%) | PCR |
| Limmathurotsakul et al (2011) | Thailand | Ubon Ratchathani | Culture | 94 / 200 (47%) | | Not done | Latex agglutination test |
| Lin et al (2011) | Taiwan | Taiwan | Culture and  PCR | 7 / 13 (53%) sampling sites by culture or PCR | | Not done | PCR |
| Mayo et al (2011) | Australia | Darwin | Culture | Not done | | 18 / 55 (33%) | PCR |
| Rattanavong et al (2011) | Lao PDR | Luangnamtha and Saravane | Culture | 198 / 900 (22%) | | Not done | Latex agglutination test |
| Trung et al (2011) | Thailand | Ubon Ratchathani | Culture and PCR | 31 / 40 (78%) by culture  33 / 40 (83%) by PCR | | Not done | PCR and latex agglutination test |

* Virulence test refers to inoculation into hamsters, guinea-pigs or rabbits, with animal pathogenicity being defined as positive for *B. pseudomallei* . Arabinose test is performed by determination of ability to assimilate L-arabinose, where *B. thailandensis* can assimilate L-arabinose and *B. pseudomallei* cannot . Specific antibody-based latex agglutination test that is positive for *B. pseudomallei,* and negative for *B. thailandensis* and other common bacteria .
