# Supplementary material for: Systematic Review and Consensus Guidelines for Environmental Sampling of Burkholderia pseudomallei
Source: PLoS Negl Trop Dis. 2013 Mar 21;7(3):e2105. doi: 10.1371/journal.pntd.0002105 (PMC3605150; doi:10.1371/journal.pntd.0002105)
Supplement: Text S1 — Data extraction form for studies that determined the presence of Burkholderia pseudomallei in the environment. (DOC) [file pntd.0002105.s003.doc]

| **A) Basic Information of The Publication or The Report** |
| --- |

Study ID |___I___I___I___I___I___I___I___| ***(Specified by admin for non-pubmed publication)***

1. Availability on Pubmed

Yes (give Study ID with “Pubmed ID”)

No (give Study ID with “running number” starting with n0000001)

Publication not available on Pubmed

Oral or poster presentation at ______________________________________________

Other, please describe __________________________________________________

2. Publication Year |___I___I___I___|  *(AD)*

3. Author list ___________________________________________________________________________

*(as presented in Pubmed)*

4. Journal _____________________________________________________________________________

5. Page _______________________________________________________________________________

6. Title of publication (or presentation) _____________________________________________________

______________________________________________________________________________________

7. Email address or contact of corresponding author ____________________________________________

______________________________________________________________________________________

8. Is this an original study describing environmental sampling to determine presence of *B. pseudomallei*

Yes (complete B and C)

No

No, but it describes additional results to the original environmental study with

Pubmed ID |___I___I___I___I___I___I___I___|

9. Is this an original study describing melioidosis in animals

Yes

No

No, but it describes additional results to the original study describing animal melioidosis with

Pubmed ID |___I___I___I___I___I___I___I___|

10. Is this an original study describing melioidosis in humans

Yes

No

No, but it describes additional results to the original study describing human melioidosis with

Pubmed ID |___I___I___I___I___I___I___I___|

*(Note: original study means “primary” or “first” report of the melioidosis or of environmental B. pseudomallei)*

| **B) Overall findings for ENVIRONMENTAL STUDIES** |
| --- |

1. Soil sampling performed  Yes  No

Positive findings in |___I___I___I___| samples in |___I___I___I___| samples tested  Not described

Positive findings in |___I___I___I___| fields in |___I___I___I___| fields tested  Not described

2. Water sampling performed  Yes  No

Positive findings in |___I___I___I___| samples in |___I___I___I___| samples tested  Not described

Positive findings in |___I___I___I___| fields in |___I___I___I___| fields tested  Not described

3. Study location and result

| Countries | Regions | District | Town | Latitude  (if available) | Longitude  (if available) | Result * |
| --- | --- | --- | --- | --- | --- | --- |
|  |  |  |  |  |  |  |
|  |  |  |  |  |  |  |
|  |  |  |  |  |  |  |
|  |  |  |  |  |  |  |
|  |  |  |  |  |  |  |
|  |  |  |  |  |  |  |
|  |  |  |  |  |  |  |
|  |  |  |  |  |  |  |
|  |  |  |  |  |  |  |

* Results include “definite, probable, possible and negative” as defined in the manuscript

4. Season at time of sampling  Dry season  Wet season  Both Wet and Dry  Not described

5. Repeat sampling (Is there any sampling point collected at more than one point of time?)

Yes  No  Not described

6. Comparing between soil positivity and other factors (more than one option is possible)

No, comparison made

Comparison of different locations, please specify______________________________________

Comparison of different seasonality, please specify____________________________________

Comparison of different depth of soil, please specify___________________________________

Comparison of different soil sampling techniques, please specify__________________________

Other comparison, please specify __________________________________________________

7. Additional information arising from this original study was presented in

Pubmed ID |___I___I___I___I___I___I___I___|

Pubmed ID |___I___I___I___I___I___I___I___|

Pubmed ID |___I___I___I___I___I___I___I___|

| **C) Methodology of Soil Sampling (Strategy of Sampling and Bacterial Isolation)** |
| --- |

1. Field use

Not Described

Described ____________________________________________________________________

*(as described in the literature if it is Pubmed publication)*

1.1. Included rice field  Yes  No  Not clearly described

1.2. Included non-rice field  Yes  No  Not clearly described

2. Sample size calculation

Not Described

Described ____________________________________________________________________

*(as described in the literature if it is Pubmed publication)*

3. Sample site selection

Not Described

Exploratory study at suspected sites based on available information; for example, areas around

households or working fields of melioidosis patients

Selected because previously positive

Convenience sampling (including sampling along roads)

Stratified random sampling

Random sampling

Random sampling using GIS program

Others, please specify___________________________________________________________

4. Sampling points per study field (e.g. Researcher may sample 1, 2, 4 or 100 sampling points per one field)

Not Described

Described |___I___I___| points per study field

5. Sampling point selection

Not Described

Fixed interval sampling at |___I___| . |___| metres apart

Random sampling

Other, please specify___________________________________________________________

6. Depth of soil sampled:

Not Described

Described, ranged from |___I___I___| to |___I___I___| centimetres

7. Amount of soil collected per sample:

Not Described

Described |___I___I___I___| gram

8. *B. pseudomallei* extraction solution

Not Described

Distilled water

Normal saline

Enrichment media, Modified Ashdown

Enrichment media, TBSS-C50

Other, please identify __________________________________________________________

9. Ratio of soil and extraction solution (wt/wt)

Not Described

Described as |___I___| : |___I___|

10. Extraction method

Not Described

Manual shaking

Vortexing

Other, please identify __________________________________________________________

11. Technique used to detect *B. pseudomallei* (more than one option is possible)

Not Described

Culture (complete Q20-24)

PCR after enrichment of *B. pseudomallei*  (complete Q20-24)

PCR without enrichment of *B. pseudomallei*

Animal inoculation

Other, please identify __________________________________________________________

12. Specimen used for *B. pseudomallei* detection (Culture, enrichment prior to PCR, PCR or animal inoculation)

Not Described

Soil plus extracted solution (No sedimentation step used, i.e. direct culture broth)

Extracted solution after sedimentation (complete Q13-19)

Other, please specify __________________________________________________________

FOR SEDIMENTATION STEP

| 13. Duration of sedimentation step  Not Described  |___I___| . |___| hours  *(20 hours is selected if described as “overnight”, 0.5 hours is selected if described as “briefly” or “short period”)*  14. Part of extracted solution used  Not Described  Upper layer of extracted solution  Other, please specify __________________________________________________________  15. Amount of extracted solution used for liquid medium  Not Described  No liquid medium used  I___I___I___|___I___I___| uL  16. Ratio of extracted solution and liquid medium used (wt/wt)  Not Described  No liquid medium used  Described as |___I___| : |___I___|    17. Liquid medium used for culture of extracted solution  Not Described  No liquid medium used  Enrichment broth, Modified Ashdown  Enrichment broth, TBSS-C50  Other, please specify __________________________________________________________  18. Amount of extracted solution used for solid medium  Not Described  No solid medium used  I___I___I___|___I___I___| uL  19. Solid medium used for culture of extracted solution  Not Described  No solid medium used  Ashdown’s agar plate  Other, please specify __________________________________________________________ |
| --- |

FOR CULTURE STEP

| 20. Duration of culture or enrichment of *B. pseudomallei*  Not Described  |___I___| days  21. Temperature of culture or enrichment of *B. pseudomallei*  Not Described  |___I___| Celcius  22. Specimen used for subculture or PCR after enrichment of *B. pseudomallei*  Not Described  Upper layer of culture broth  Other, please identify __________________________________________________________  23. Volume used for subculture or PCR after enrichment of *B. pseudomallei*  Not Described  |___I___I___I___| microlitre  24. Agar used for subculture  Not Described  Ashdown’s agar plate  Other, please specify __________________________________________________________  25. Quantitative count  No  Yes, please describe the result __________________________________________________  26. Method of confirmation of *B. pseudomallei* isolated (more than one option is possible)  Not Described  Basic microbiological test (typical colony morphology, Gram stain, oxidase test)  Automated system (e.g. Vitek)  API20NE  Arabinose test reference Pubmed ID |___I___I___I___I___I___I___I___|  Latex agglutination reference Pubmed ID |___I___I___I___I___I___I___I___|  PCR reference Pubmed ID |___I___I___I___I___I___I___I___|  Genotyping such as MLST reference Pubmed ID |___I___I___I___I___I___I___I___|  Other, please specify ___________________________________________________________  27. Any other comments __________________________________________________________________ |
| --- |

| **D) Methodology of Water Sampling (Strategy of Sampling and Bacterial Isolation)** |
| --- |

1. Field use

Not Described

Described ____________________________________________________________________

*(as described in the literature if it is Pubmed publication)*

2. Amount of water collected per sample:

Not Described

Described |___I___I___I___| ml

3. Technique used to detect *B. pseudomallei* (more than one option is possible)

Not Described

Culture

PCR after enrichment of *B. pseudomallei*

PCR without enrichment of *B. pseudomallei*

Animal inoculation

Others, please specify __________________________________________________________

4. Specimen for *B. pseudomallei* detection

Not Described

Water

Supernatant after suspension  Deposit after centrifugation

Filter 0.22 micron Filter 0.45 micron

Filter unknown size

Other, please specify __________________________________________________________

5. Liquid medium used for culture of extracted solution

Not Described  No liquid medium used

Enrichment broth, Modified Ashdown  Enrichment broth, TBSS-C50

Other, please specify __________________________________________________________

6. Solid medium used for culture of extracted solution

Not Described

No solid medium used

Ashdown’s agar plate

Other, please specify __________________________________________________________

7. Quantitative count

No

Yes, please describe the result____________________________________________________
