# Supplementary material for: Systematic Review and Consensus Guidelines for Environmental Sampling of Burkholderia pseudomallei
Source: PLoS Negl Trop Dis. 2013 Mar 21;7(3):e2105. doi: 10.1371/journal.pntd.0002105 (PMC3605150; doi:10.1371/journal.pntd.0002105)
Supplement: Text S2 — Questionnaire on the detection of environmental Burkholderia pseudomallei. (DOC) [file pntd.0002105.s004.doc]

**Questionnaire on the detection of environmental *Burkholderia pseudomallei* (29 July 2011)**

Dear Colleague

We aim to develop consensus guidelines on methodology for the detection of environmental *Burkholderia pseudomallei*. An important objective is to make this affordable to facilitate uptake worldwide, and so we have based our method on simple qualitative culture.

We have developed a short questionnaire following a systematic review of the relevant literature, in which we hope we have identified those areas where opinion may differ over methodology. We hope that you can find the time to complete this and in so doing assist the melioidosis community in reaching a consensus. We welcome disagreement and debate, since we are keen to be sure that any recommendations that arise will be both useful and inclusive. Please note that these recommendations are specifically for the yes/no detection of *B. pseudomallei*, and not all of these may be applicable to studies with more complex specific objectives such as the presence of the organism at different depths in soil, or the relationship between the organism and different habitats.

If you return a completed questionnaire to us, you will be listed as a member of the working group for this study on the related publication. We would be grateful if you would return the completed the questionnaire to Direk Limmathurotsakul (direk@tropmedres.ac) within 15 days.

Thank you in advance for your help.

Yours sincerely,

Sharon and Direk

Sharon Peacock

Professor of Clinical Microbiology

University of Cambridge, UK

Direk Limmathurotsakul

Deputy Head of Microbiology

Mahidol-Oxford Tropical Medicine Research Unit, Thailand

**QUESTIONNAIRE:** Published and proposed methodology to determine “**the presence of *B. pseudomallei* in the environment”**

First Name & Initial . Surname Degree

Affiliation Email

| Question | Published evidence  (supporting notes below) | Our suggested recommendation | **Your opinion** |
| --- | --- | --- | --- |
| *Sampling strategy* | |  |  |
| Sample size calculation | Not stated and often small | Sample size calculation should be presented | |___| I agree  |___| I do not agree because: |
| Choice of sampling site | Variable, including random site selection and practical considerations (e.g. sampling at points along a main road) | - For pilot studies in areas not sampled previously, choose areas most likely to be positive - For sampling over a wider area, random site selection using GPS programme | |___| I agree with both  |___| I do not agree because |
| Number of samples taken per site | Ranged from 2 to 100 points from a single defined sampling site | - 100 points per site - Use a fixed interval sampling grid | |___| I agree with both  |___| I do not agree because: |
| Distance between sampling points in a single site | 1 to 5 metres, or not stated | If no prior information available for *B. pseudomallei* distribution in test area, take samples at a distance of 2.5 to 5 metres apart | |___| I agree  |___| I do not agree because: |
| Soil sampling depth | Ranged from 0 to 90 cm | 30 cm | |___| I agree  |___| I do not agree because: |
| Weight of each soil sample | Ranged from 2 to 1,000 gram of soil | - 10 gram of soil - Place into universal tube | |___| I agree with both  |___| I do not agree with one or both because: |
| Temperature during transportation of sample to laboratory | Variable, including at room temperature and at refrigerated temperature | At ambient temperature | |___| I agree  |___| I do not agree because: |
| *Extraction of B. pseudomallei from soil* | | | |
| Solution used to extract *B. pseudomallei* from soil | Distilled water, normal saline or enrichment media | Threonine-basal salt plus colistin 50 mg/L (TBSS-C50 broth) | |___| I agree  |___| I do not agree because: |
| Ratio of soil and extraction solution (wt/vol) | Ranged from 2:1 to 1:10 | 1:1  (10 gram soil/10 ml TBSS-C50) | |___| I agree  |___| I do not agree because: |
| Mixing of soil and extraction solution | Variable, including manual shaking, vortexing or orbital shaker for 1 minute to 24 hours | Vortexing for 30 seconds | |___| I agree  |___| I do not agree because: |
| *Detection of B. pseudomallei* | |  |  |
| Detection technique | Culture, PCR or animal inoculation | Culture | |___| I agree  |___| I do not agree because: |
| Protocol for culture | Variable | Incubate the specimen (universal tube with 10g of soil + 10ml TBSS-C50) for 48 hours | |___| I agree  |___| I do not agree because: |
| Temperature of incubator | Variable, ranged from 37 to 42°C | 42°C | |___| I agree  |___| I do not agree because: |
| Protocol for Subculture | Variable | - Subculture 10 uL of supernatant onto half an Ashdown agar plate - Incubate plate and examine every 24 hours for 4 days | |___| I agree with both  |___| I do not agree because: |
| Identification of *B. pseudomallei* | Variable | - Basic microbiological tests (typical colony morphology, Gram stain, positive oxidase test, inability to assiminate arabinose, resistance to gentamicin and colistin with susceptibility to co-amoxiclav) - Use confirmatory test (API20NE, Vitek system, specific latex agglutination test or PCR assay) | |___| I agree with both  |___| I do not agree because: |
| *Describing methods and reporting findings* | | |  |
| After publication deposit raw data to website | Website to deposit and share raw data is not available | After publication deposit raw data to website [www.melioidosis.info](http://www.melioidosis.info/) | |___| I agree  |___| I do not agree because: |
| GPS location of study site | Variably reported | Describe in methods, and after publication deposit raw data to website [www.melioidosis.info](http://www.melioidosis.info/) | |___| I agree  |___| I do not agree because: |
| Positivity rate in each study site and pattern of positivity in each study site | Variably reported | Describe in methods, and after publication deposit raw data to website [www.melioidosis.info](http://www.melioidosis.info/) | |___| I agree with both  |___| I do not agree because: |
| Soil type and history of land use | Variably reported | Describe in methods, and after publication deposit raw data to website [www.melioidosis.info](http://www.melioidosis.info/) | |___| I agree with both  |___| I do not agree because: |
| Sampling time and weather at sampling time point (e.g. rainfall, season) | Variably reported | Describe in methods and after publication deposit raw data to website [www.melioidosis.info](http://www.melioidosis.info/) | |___| I agree with both  |___| I do not agree because: |
| *Your comments* |  |  |  |
| Space for your comments |  | | |

**NOTES TO ACCOMPANY QUESTIONNAIRE**

**Choosing the sampling site and design** The most appropriate sampling strategy will depend on the objectives of the study, and whether any information is already available for the geographical area to be sampled. To determine the presence of environmental *B. pseudomallei* in a given area, prior information should be sought of possible melioidosis cases. If suspected or definite cases of melioidosis are identified, then sampling could initially be targeted to an area close to their residence or workplace. In the absence of such clinical information, a pilot study could be performed to obtain preliminary data, followed (if resources allow) by a more extensive survey using GPS programmes to randomly identify several locations within a study area. The use of a fixed interval grid is cheap and simple, and facilitates the comparison of data between studies.

**Number of samples** Taking insufficient soil samples from a designated sampling site runs the risk of a false negative result. This is because saprophytic bacteria exist in aggregates, which results in the presence of environmental hot spots and may give rise to intervening areas that are apparently negative for a specific bacterium. This has been shown to be the case for *B. pseudomallei*. Because of this, random sampling methods using a low sample size may be associated with a low power of detection and a high false negative rate (type II error). This can be avoided by increasing the number of samples taken.

**Distance between samples** The presence of environmental hot spots leads to an effect described by the term ‘spatial autocorrelation’, which influences the distance required between each sampling point. What this means in practice is that sampling points adjacent to each other are more likely to yield the same result (e.g. a sample next to a negative sample is likely to be negative). The distance over which counts of a given environmental bacterium are related (range of spatial autocorrelation) can be defined using a geostatistical tool called the semivariogram. Ideally, the effect of spatial autocorrelation would be factored in to the sampling strategy for *B. pseudomallei*, but this value is likely to vary between and possibly within countries, and it is not practical to define this prior to formal sampling in most settings. Studies in Thailand suggest that the distance between samples should be between 2.5 and 5m apart, but it is uncertain whether this applies elsewhere and a study in Australia failed to find autocorrelation. Given the relative lack of data on the optimal distance between samples we suggest that sampling be performed 2.5 to 5m apart, accepting that this is somewhat arbitrary and that a region-specific semivariogram could be calculated based on the first pilot study in a given area.

**Soil sampling: quantity, sampling depth and transport to the laboratory** The quantity of soil collected per sample has varied markedly between published studies. No comparative studies have been performed to determine the optimal quantity, and there is no evidence that collecting a greater weight of soil is associated with a higher sensitivity. We suggest taking a sample weight of 10 grams per sample based on practicality and ease of methodology. Our recommended depth at which soil should be taken is 30 cm. This is based on published evidence that the proportion of samples culture positive for *B. pseudomallei* is higher at 30 cm than at shallower depth, and comparable to deeper than 30 cm. This finding is consistent in Australia, northeast Thailand, Taiwan and Brazil. Based on a report that survival of *B. pseudomallei* decreases at low temperature, transportation of soil samples to the laboratory should be maintained at ambient temperature.

**Extraction of bacteria from soil, and detection and identification of *B. pseudomallei.*** The optimal ratio of soil to extraction solution, mixing technique and sedimentation time are not known. Selective broths have been compared in both laboratory and field settings, with the latter study determining that TBSS with colistin was the best selective enrichment broth of the three tested.

**Data sharing.** The consortium is developing a website ([www.melioidosis.info](http://www.melioidosis.info/)) where complete data from sampling studies can be deposited with the assistance of a curator (DL). This will be used to build a global map of the distribution of environmental *B. pseudomallei*, as well as those places where melioidosis has been acquired by humans and animals. The website will also provide downloadable protocols describing methodology for soil sampling and culture.
