# Supplementary material for: Systematic Review and Consensus Guidelines for Environmental Sampling of Burkholderia pseudomallei
Source: PLoS Negl Trop Dis. 2013 Mar 21;7(3):e2105. doi: 10.1371/journal.pntd.0002105 (PMC3605150; doi:10.1371/journal.pntd.0002105)
Supplement: Text S3 — Standard Operating Procedure (SOP): simplified method for the isolation of Burkholderia pseudomallei from soil. (DOC) [file pntd.0002105.s005.doc]

**Standard Operating Procedure (SOP)**

**Simplified method for the isolation of *Burkholderia pseudomallei* from soil**

**Compiled by: Ms. Vanaporn Wuthiekanun (VW) and**

**Dr. David Dance (DD)**

**on behalf of the**

**Detection of Environmental *Burkholderia pseudomallei* Working Party (DEBWorP)**

# *1. Background*

Detection of environmental *B. pseudomallei* indicates a potential risk for melioidosis, and is important for the development of a global risk map. Here, we describe a simple method to detect *B. pseudomallei* using direct soil culture in enrichment broth. Based on statistical considerations described in the accompanying review, a minimum of 100 sampling points is suggested. This method is currently used at the Mahidol-Oxford Tropical Medicine Research Unit Thailand, but variations are possible as described in the review and consensus guidelines.

# *2. Objective*

To determine the presence of *B. pseudomallei* in an area of around 50x50 sq meters.

***3. Soil sampling***

**3.1 Equipment & consumables**

- Shovel
- Disposable latex gloves or equivalent
- Surgical masks
- Rubber boots
- 70% alcohol spray
- Paper towels
- Sterile containers (e.g 25 ml plastic universal containers, ziplock plastic bags for human milk etc. according to local availability)
- String and stakes
- Weighing scales if soil to be added on site
- Labelling pens if tubes not pre-labelled
- Insulated container (e.g. polystyrene box, picnic box etc.)
- Tape measure (at least 5 m and preferably longer)

**3.2 Preparation**

3.2.1 Select location of the sampling site as described in the consensus guidelines.

3.2.2 Divide the sampling site into a 10 x 10 grid of squares, each 5 m x 5 m (total 100 squares) by measuring with tape measure and marking with stakes and strings.

3.2.3 Define and mark the centre of each square using tape measure and/or strings, where the sample will be collected.

**3.3 Soil sampling**

3.3.1 Wear protective gear including gloves, boots, and possibly masks, according to local risk assessment.

3.3.1 In order to avoid cross-contamination, clean all instruments with clean water (for example, bottled water) to remove all visible dirt or debris, spray with 70% alcohol and allow to air dry, then wipe off any residual moisture with a clean tissue prior to sampling

3.3.2 Either

a.) Dig a hole using a clean, small metal gardening shovel with a pointed end to collect a soil sample at depth of 30 cm,

or

b.) Collect a soil sample at depth of 30 cm using an auger.

3.3.3 Transfer about 20 to 40 g soil to sterile containers (alternatively 10 g samples may be weighed directly into sterile 25 ml universal tubes if preferred).

3.3.4 Cap and label the container, and place it in an insulated container in the shade.

3.3.5 Transport samples to the laboratory at ambient temperature without exposure to direct sunlight and process as quickly as possible.

***4. Laboratory processing of soil specimen***

**4.1 Equipment and facilities**

- Scales
- Incubator set at 40°C (Incubator set at 37°C is optional)
- Class II (or Class I) Biological Safety Cabinet (BSC)
- Adjustable pipette (10 μl)
- Vortex mixer
- Racks
- BSL3 Laboratory (subject to local regulations and derogations)

**4.2 Media and reagents**

- Selective agar (Ashdown agar [ASH] – see Appendix 1)
- Selective broth (Threonine-basal-salt-solution [TBSS-C50] – see Appendix 1)
- Columbia agar or Mueller-Hinton agar (for screening of antibiotic susceptibility)
- *B. pseudomallei*-specific latex agglutination reagent (available for purchase from [lek@tropmedres.ac](mailto:lek@tropmedres.ac) or [sura_wng@kku.ac.th](mailto:sura_wng@kku.ac.th))

**4.3 Other consumables**

- Sterile 25 ml universal tubes
- Sterile 10 ml pipettes
- Pipette aid
- Sterile tips for adjustable pipette
- Sticky labels
- Sterile loops
- Personal Protective Equipment according to local safety guidelines

**4. 4 Culture method**

- - 1. Weigh 10 gram of soil, using scales, in a sterile 25 ml universal tube.
    2. Add 10 ml of threonine-basal salt solution plus colistin 50 mg/L (TBSS-C50) into the universal tube.
    3. After ensuring that the lid is firmly replaced, vortex the universal tube for 30 seconds.
    4. Incubate the universal tube at 40°C in air for 48 hours.
    5. Subculture 10 μl of the upper layer of the medium onto an Ashdown agar plate in a BSC, streaking to achieve single colonies.
    6. Incubate the Ashdown agar plate at 40°C in air.
    7. Examine the Ashdown agar plate daily for 7 days in a BSC for colonies suspected to be *B. pseudomallei* based on the following characteristics:

At 24 hours colonies are often pinpoint, clear and pale pink, changing to become pinkish-purple, flat, and slightly dry in the next 2 days. The most characteristic feature of *B. pseudomallei* is its metallic sheen, and the usual progression to dry and wrinkled colonies at 96 hours. Colonial variants are common, and include smooth and shiny, mucoid or dry, or different shades of purple.[^3^](#_ENREF_3)

- - 1. Any bacterial colony suspected to be *B. pseudomallei* should be identified by the following methods.

***5. Identification of Burkholderia pseudomallei***

**5.1 Latex agglutination test**

Initial screening of suspect colonies from Ashdown agar is undertaken by latex agglutination using latex particles coated with monoclonal antibodies specific for the 200-kDa exopolysaccharide of *B*. *pseudomallei*.[^4^](#_ENREF_4) Anyone interested in obtaining this reagent should contact VW at the Mahidol-Oxford Tropical Medicine Research Unit ([lek@tropmedres.ac](mailto:lek@tropmedres.ac) or [sura_wng@kku.ac.th](mailto:sura_wng@kku.ac.th)).

5.1.1 Use a toothpick to pick a suspected colony and mix the bacterium with 5-10 μl latex reagent on a glass slide.

5.1.2 Gently rock the slide, to keep the fluid suspension in constant movement for 2 minutes.

5.1.3 Observe for positive agglutination: fine, but readily discernible granularity against clear background.

5.1.4 Confirm each batch of tests with a positive (e.g. *B. pseudomallei* NR 8071 - see [http://www.beiresources.org](http://www.beiresources.org/)) and negative (e.g. *B. thailandensis* ATCC 700388) control strain according to local availability.

**5.2 Screen for antimicrobial susceptibility**

5.2.1 Select one latex positive colony from ASH (or all colony morphology types if more than one confirmed by latex) and streak onto half a Columbia agar plate to which apply 10 μg colistin (CT10) and 30 μg amoxicillin-clavulanate (AMC 30) antibiotic discs.

5.2.2 Incubate overnight in air at 37°C.

5.2.3 *B. pseudomallei* will be resistant to colistin (no zone) and have a clear zone around amoxicillin-clavulanate and should have a metallic sheen on Columbia agar. *Burkholderia cepacia* is resistant to amoxicillin-clavulanate and the colonies may appear yellow or light brown on Columbia agar.

**5.3 Definitive identification**

Any organism that has a colonial appearance typical of *B. pseudomallei* on Ashdown agar and is resistant to colistin and susceptible to co-amoxiclav can be presumptively identified as *B. pseudomallei* if it is positive for latex agglutination. Any organism that has a colonial appearance similar to *B. pseudomallei* on Ashdown agar and is resistant to colistin and susceptible to co-amoxiclav is likely to be *B. thailandensis* if it is negative for latex agglutination. We recommend that environmental isolates presumptively identified as *Burkholderia* species for the first time in a given country or region should be referred to a national or international reference laboratory for definitive identification.

Several methods are applicable for definitive identification of isolates presumptively identified as *B. pseudomallei* or *B. thailandensis*, including biochemical test kits such as the API 20NE or molecular identification tests depending on what is available locally. These are described in more detail in the review and consensus guidelines.

Please note that typical API 20 NE (also called 20 NFT) (BioMerieux, Durham, N.C.), profiles for *B. pseudomallei* are 1156577, 1556577 or 1156576. Since this kit was designed for clinical rather than soil isolates, there are no profiles for *B. thailandensis* in its database, but *B. thailandensis* gives results similar to *B. pseudomallei* with the exception of positive arabinose assimilation (i.e. 1157577, 1557577 or 1157576)*.* With all these commercial systems, *Burkholderia pseudomallei* may sometimes be misidentified as *Burkholderia cepacia*, *Pseudomonas fluorescens*, *Pseudomonas aeruginosa*, or *Chromobacterium violaceum.*

***6. References:***

1. Limmathurotsakul D, Wuthiekanun V, Chantratita N, et al. *Burkholderia pseudomallei* is spatially distributed in soil in northeast Thailand. PLoS Negl Trop Dis 2010;4:e694.

2. Limmathurotsakul D, Wuthiekanun V, Amornchai P, Wongsuwan G, Day NP, Peacock SJ. Effectiveness of a Simplified Method for Isolation of *Burkholderia pseudomallei* from Soil. Applied and environmental microbiology 2012;78:876-7.

3. Chantratita N, Wuthiekanun V, Boonbumrung K, et al. Biological relevance of colony morphology and phenotypic switching by *Burkholderia pseudomallei.* J Bacteriol 2007;189:807-17.

4. Anuntagool N, Naigowit P, Petkanchanapong V, Aramsri P, Panichakul T, Sirisinha S. Monoclonal antibody-based rapid identification of *Burkholderia pseudomallei* in blood culture fluid from patients with community-acquired septicaemia. J Med Microbiol 2000;49:1075-8.

***Appendix 1***

***Media preparation***

***THREONINE BASAL SALT SOLUTION (TBSS-C50)***

1) SOLUTION A

| **Ingredients** | **Amount** | **Manufacturer/Catalog no.** |
| --- | --- | --- |
| H_3_PO_4_ 85% | 2.306 ml | Sigma/P-6560 |
| FeSO_4_.7H_2_O | 0.556 g | Merck/1.03965 |
| ZnSO_4_.7H_2_O | 0.297 g | Merck/8883 |
| CuSO_4_.5H_2_O | 0.0218 g | Merck/2790 |
| MnSO_4_.H_2_O | 0.125 g | Univar/309 |
| Co(NO_3_)_2_.6H_2_O | 0.030 g | BDH/3714260 |
| Na_2_MoO_4_.2H_2_O | 0.030 g | Univar/360 |
| H_3_BO_3_ | 0.062 g | Hopkin&Williams/227800 |
| Distilled water | 1000 ml |  |

**Procedure**

1.1 Mix all the above ingredients in a 1 litre bottle on a hot plate with a magnetic stirrer until all are dissolved.

1.2 Sterilise by autoclaving at 121°C for 20 minutes.

2) BASE

| **Ingredients** | **Amount** | **Manufacturer/Catalog no.** |
| --- | --- | --- |
| KH_2_PO_4_ | 0.451 g | Sigma/P5379 |
| K_2_HPO_4_ | 1.730 g | Merck/1.05104 |
| MgSO_4._7H_2_O | 0.123 g | Sigma/M5921 |
| CaCl_2_ .2H_2_O | 0.0147 g | Merck/1.02382 |
| NaCl | 10 g | BDH/102415K |
| Nitrilotriacetic acid | 0.200 g | BDH/29338 |
| Solution A | 20 ml |  |
| Distilled water | 900 ml |  |

**Procedure**

2.1 Mix all the above ingredients in a 1-litre bottle.

2.2 Adjust pH to 7.2 with 1N KOH.

2.3 Sterilise by autoclaving at 121°C for 20 minutes.

3) L-THREONINE SOLUTION

| **Ingredients** | **Amount** | **Manufacturer/Catalog no.** |
| --- | --- | --- |
| L-Threonine | 5.956 g | Merck/1.08411 |
| Distilled water | 100 ml |  |

**Procedure**

- 1. Mix the above ingredients in a 100 ml bottle.
  2. Sterilise by filtration through 0.20 μm filter (e.g. Sartorius 16534 minisart 0.20 µm CE non-pyrogenic sterile-EO, 0.20 μm Millipore filter).

**4) TBSS-C50**

**Procedure**

4.1 Add 100 ml of L-Threonine solution to 900 ml of the base medium (final concentration of L-Threonine 0.05M).

4.2 Add Colistin Sulphomethate Sodium BP to a final concentration of 50 mg/l (e.g. 1ml of Colistin 1x10^6^ unit/ml dissolved in 1.6 ml sterilised water).

# *ASHDOWN AGAR (ASH)*

| **Ingredients** | **Amount** | **Manufacturer/Catalog no.** |
| --- | --- | --- |
| Tryptone soya broth | 10 g | Oxoid/CM129 |
| Agar | 15 g | Oxoid/LP0011 |
| Glycerol | 40 ml | BDH/24388.320 |
| Crystal violet 0.1% * | 5 ml | Merck/1.15940.0100 |
| Neutral red 1% | 5 ml | Merck/1.01369.0025 |
| Distilled water | 950 ml |  |

**Procedure**

1. Mix all ingredients in a bottle.

2. Autoclave at 121°C for 15 min.

3. Cool to 50°C, add gentamicin to a final concentration of 4 mg/l

4. Dispense the agar into petri-dishes.

**Note** * The crystal violet solution 0.1% should be incubated at 37°C for two weeks before being used in order to ensure optimal coloration.

***Appendix 2***

***Media Quality Control***

1. Sterility test: a representative sample of each lot/batch of medium should be incubated for 2 days in air at 35-37°C. Use 2 agar plates or 2 tubes of broth per 1 litre of each batch of medium. If there is no growth after 2 days, then the batch is ready to be used.

2. Growth performance: test the ability of the medium to support the growth of *B. pseudomallei* NR 8071 (see [http://www.beiresources.org](http://www.beiresources.org/)) as follows:

- 1. Touch 5-7 colonies of a pure *B. pseudomallei* culture (e.g. a 48 hour ASH agar culture or 18-24 hour Columbia agar culture) with a cotton swab.
  2. Emulsify the colonies in 3 ml of Normal Saline (0.85% w/v NaCl) and adjust the suspension to match a 1.0 McFarland turbidity standard (Densimat or spectrophotometer set at 360 nm, ABS, tungsten, equal to 0.5), to get an approximate concentration of 1x10^8^ CFU/ml.
  3. Make serial 10-fold dilution of the 1.0 MacFarland suspension for 6 dilutions (10^-1^, 10^-2^, 10^-3^, 10^-4^, 10^-5^, 10^-6^ dilutions).
  4. Drop 10 µl of the 10^-4^, 10^-5^, 10^-6^ dilutions onto 1/3 of an Ashdown’s agar plate or into 10 ml TBSS-C50 broth in duplicate.
  5. Incubate broth for 2 days in air at 40°C before subculturing 10 μl from the surface layer of broth onto 1/3 of an Ashdown’s agar plate
  6. Incubate all agar plates in air at 40°C for 4 days
  7. If all plates give a good growth of organism for all dilutions, then the media are ready to be used.

***Appendix 3***

***Safety considerations***

All procedures should follow local safety rules and national regulations and should be subject to local risk assessment. The following guidance would normally be applicable.

1. All inspection of culture plates and manipulations of microbiological organisms should be carried out in a biological safety cabinet (Class I or II).
2. Once *B. pseudomallei* is suspected, all subsequent laboratory procedures should be carried out at biosafety level 3 (BSL3).
3. Protective gloves and boots should be worn during soil sampling. If the generation of aerosols is considered likely then consideration can also be given to the use of masks. Protective clothing, including long sleeves and sun hats, and sunblock cream should also be worn if necessary.
4. Other potential risks of sampling a given site that may need to be considered include power cables, concealed holes in the ground, unexploded ordnance, unstable masonry, traffic etc.
5. During environmental sampling it is preferable to work in teams of at least two people.
